# Management of isolated coronal shear fractures of the humeral capitellum with Herbert screw fixation through anterolateral approach

**DOI:** 10.1186/s12891-018-2024-8

**Published:** 2018-04-05

**Authors:** Tengbo Yu, Hao Tao, Fenglei Xu, Yanling Hu, Chengdong Zhang, Guangjie Zhou

**Affiliations:** 1 Department of Orthopaedic Surgery, Affiliated Hospital of Qingdao University, Qingdao, Shandong 266000 People’s Republic of China; 2 Department of Radiology, Affiliated Hospital of Qingdao University, Qingdao, Shandong 266000 People’s Republic of China; 3 Department of Orthopaedic Surgery, Affiliated Hospital of Qingdao University, 59 Haier Road, Qingdao City, 266003 Shandong Province China

**Keywords:** Capitellum, Fracture, Internal fixation, Anterolateral approach, Herbert screw

## Abstract

**Background:**

Due to the intraarticular and complex nature of the coronal shear fracture of the humeral capitellum and its rarity, it has been difficult to formulate a universally accepted method of surgical management. The purpose of this study is to retrospectively evaluate the clinical outcomes of 15 patients with isolated coronal shear fractures of the capitellum treated by Herbert screw fixation through anterolateral approach, and to address the safety and tips for this surgical procedure.

**Methods:**

This retrospective study included 15 isolated coronal shear fractures of the capitellum without posterior involvement, which were classified according to the Dubberley classification as 11 type 1A fractures and 4 type 3A fractures. All fractures were treated with Herbert screws fixation via the anterolateral approach. Clinical and radiographic evaluation was performed regularly, with a mean follow-up of 29 months.

**Results:**

The mean operative time was 81 min. There were no wound healing problems or infection. One incomplete posterior interosseous nerve injury occurred, which recovered soon without residual compromise. All fractures healed well. At the final follow-up, the average range of motion was 134°in flexion-extension and 172°in supination-pronation. There was no significant difference between the affected and the unaffected elbows with regard to motion in flexion-extension or flexion-extension. The average Mayo Elbow Performance Index Score was 93 with 11 excellent and 4 good. No evidence of avascular necrosis, posttraumatic osteoarthritis, or heterotrophic ossification was found.

**Conclusion:**

Open reduction and internal fixation using Herbert screws through a anterolateral approach is a reliable and effective treatment for coronal shear fractures of capitellum, and able to achieve stable fixation and restoration of a functional range of motion.

## Background

Fractures of the humeral capitellum are rare injuries, accounting for nearly 1% among all elbow fractures [1]. These fractures are usually a result of axial loading to the capitellum and occasionally to the trochlea transmitted through the radial head.

Treatment strategies for these injuries have evolved from conservative management to surgical management. Currently, open reduction and internal fixation with an aim to provide stable and congruent joint has been regarded as preferred treatment [2–8], whereas the intraarticular and complex nature of these fractures makes optimal surgical exposure and fixation method debatable. For coronal shear fracture of capitellum without involvement of posterior aspect, the most commonly used approach is the lateral approach of elbow joint [2–8]. Though favorable outcomes have been reported, exposure of the radiocapitellar compartment and visualization of the trochlea and medial articular extension is inadequate through this approach. Several studies have adopted the anterolateral approach to the elbow joint to treat this type of fracture, which is superior to the lateral approach for exposure range of the anterior aspect of the elbow joint [9–11]. As regard to articular surface reconstruction, various implants including Kirschner wires, headleass compression screws, Herbert screws, minifragment screws, and bioabsorbable implants have been adopted. Herbert screw fixation is a good option due to excellent compression at the fracture site, stable fixation, and nonprominence of the implant intra-articularly [12].

Given the intraarticular and complex nature of the coronal shear fracture of the humeral capitellum and its rarity, it has been difficult to formulate a universally accepted method of surgical management. The purpose of this study is to present the clinical outcomes of a retrospective study of 15 cases with isolated coronal shear fractures of the capitellum treated by Herbert screw fixation through anterolateral approach, and to address the safety and tips for this surgical procedure. We hypothesized that open reduction and Herbert screws fixation through anterolateral approach is a reliable and effective management for coronal shear fractures of capitellum.

## Methods

### Patients

From January 2009 to June 2015, 18 consecutive patients with isolated coronal shear fractures of the capitellum without posterior condyle involvement were treated with open reduction and Herbert screw fixation through the anterolateral approach within 2 weeks after the injury. Two patients were lost to follow-up, and 1 died of unrelated illness. This study was approved by the Institutional Review Board of the affiliated hospital of Qingdao University, and 15 patients consented to participate in the study. Nine patients were female, and 6 were male. The mean age (and standard deviation) was 42 ± 13 years (range, 19 to 64 years). Ten patients occurred after a fall, and 5 occurred in road traffic accidents. The mean time from presentation to surgical treatment was 4 ± 1 days (range, 1 to 7 days). The mean duration of follow-up was 29 ± 4 months (range, 24 to 36 months) (Table).

Plain radiographs were performed routinely. All patients underwent computed tomography (CT) scans with a 3D reconstruction for better definition of the fracture line and to rule out associated injuries, such as the coronoid process fractures, a dislocation or injury to the radial head, epicondylar avulsion fractures or elbow dislocations. Only isolated coronal shear fractures were included in this study.

Fractures in this study were classified according to the Dubberley classification system [2].Type 1 is a fracture involving primarily the capitellum with or without the lateral trochlear ridge. Type 2 is a fracture involving the capitellum and the trochlea as one piece. Type 3 is a fracture involving both the capitellum and the trochlea as separate fragments. These fractures were further classified as type A and type B based on the absence or presence of posterior condylar comminution. All fractures in the current series were coronal shear fractures without posterior comminution. Eleven patients sustained type 1A fracture, 4 were type 3A.

### Surgical technique

All the patients were administered a brachial plexus anesthesia and placed in the supine position with a tourniquet on the upper arm. Varus and valgus stress examination under anesthesia was performed to rule out concomitant ligamentous injury.

**Fig. 1:**
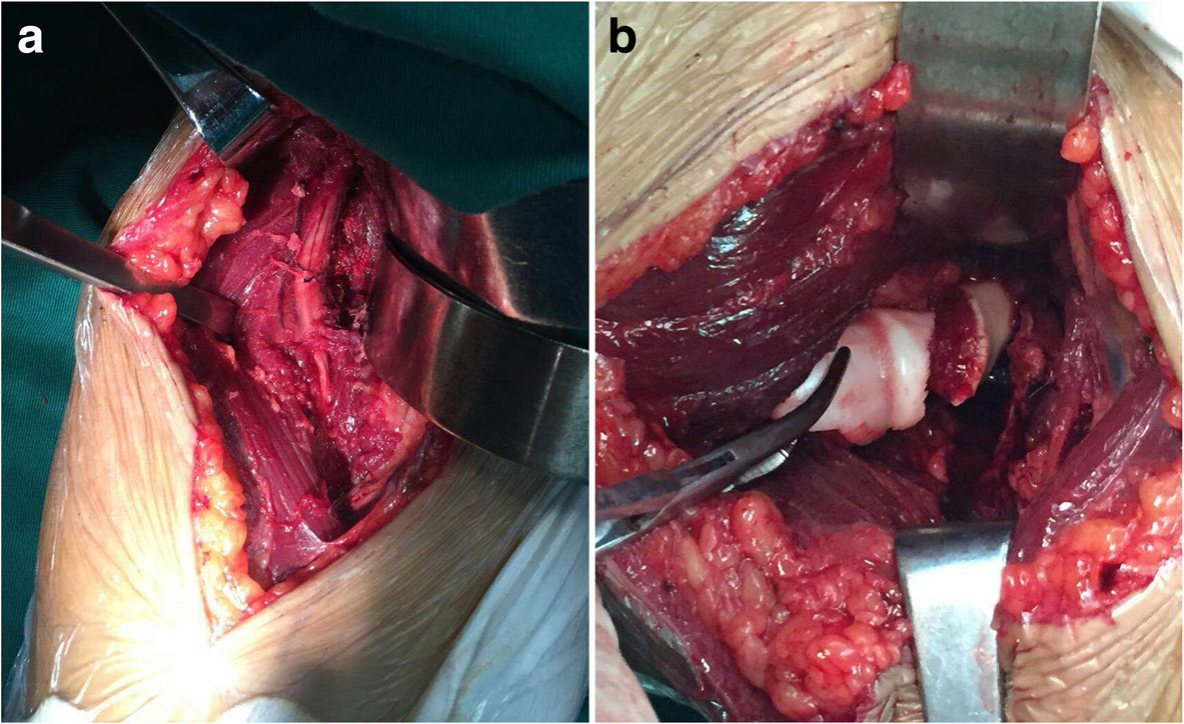
Dissection of anterolateral approach to the elbow joint. **a**: The dissection of radial nerve. **b**: The exposure of fracture fragment

A curved incision began 5 cm above the elbow flexion crease in the supinated forearm, and followed the lateral border of the biceps distally, but curved laterally at the elbow joint level to avoid crossing a flexion crease at 90°. Then it extended distally in the forearm along the medial border of brachioradialis. The interval was made between the brachialis and brachioradialis. The forearm lateral cutaneous nerve need to be protected in the superficial plane. In the deeper plane, the radial nerve need to be identified and protected (Fig. 1a). The brachioradialis and the radial nerve were retracted laterally and the biceps medially to expose the anterior capsule of the elbow joint. The capsule was incised to expose the capitellum (Fig. 1b).

The fracture site was debrided by removing blood clots, loose pieces of bone, and any interposed tissue. Saline irrigation was used to achieve greater clarity. The fracture was reduced by matching the articular fracture lines. Provisional fixation was performed with 2 or 3 guidewires for the Herbert screw. The guidewires were passed across the fracture site where the planned screw track was to be inserted. After anatomic reduction was confirmed with fluoroscopy, Herbert screws were inserted over the guidewires in anterior to posterior direction to achieve definitive fixation. The screws were buried beneath the articular surface. Upon fixation, the elbow was made to go through the full flexion-extension and rotation arc to check for the stability of fixation. Final reduction and position of the implant was checked with fluoroscopy. The closure of the wound was done in layers over a drain.

### Postoperative care

A long arm posterior plaster splint was applied routinely with the elbow at approximately 90° of flexion, which was kept for 2 week. Active range of motion (ROM) was started when the splint was removed.

Operation time, wound healing complication and nerve injury were recorded. Clinical and radiographic evaluation was performed postoperatively at approximately 1, 2, 3, 6, 12 months. Then patients were called back for evaluation for the purposes of this study. At each follow-up, Pain, ROM and stability of the elbow joint was assessed by clinical examination, which enabled calculation of the Mayo Elbow Performance Index (MEPI) Score. The MEPI consists of four parts: pain (with a maximum score of 45 points), ulnohumeral motion (20 points), stability (10 points) and the ability to perform five functional tasks (25 points). If the total score is included between 90 and 100 points, it can be considered excellent; between 75 and 89 points, good; between 60 and 74 points, fair; and less than 60 points, poor. In addition, radiographic examination was performed to evaluate the status of the bony union, heterotopic ossification, incidence of posttraumatic osteoarthritis and avascular necrosis.

#### Statistical analysis

The paired t-test was used for statistical comparisons with regard to ROM between the affected and the unaffected elbow with SPSS for Windows 15.0 (SPSS, USA). Differences were considered to be significant if *p* < 0.05.

## Results

The mean operative time was 81 ± 12 min (range, 65 to 105 min). There were no wound healing problems and infection. One patient sustained incomplete posterior interosseous nerve palsy, who presented with extension deficit of his ring finger and little finger at the metacarpophalangeal joint level. It recovered completely in 4 weeks without residual compromise. All fractures healed well in their normal anatomic position as seen on radiographs.

**Fig. 2:**
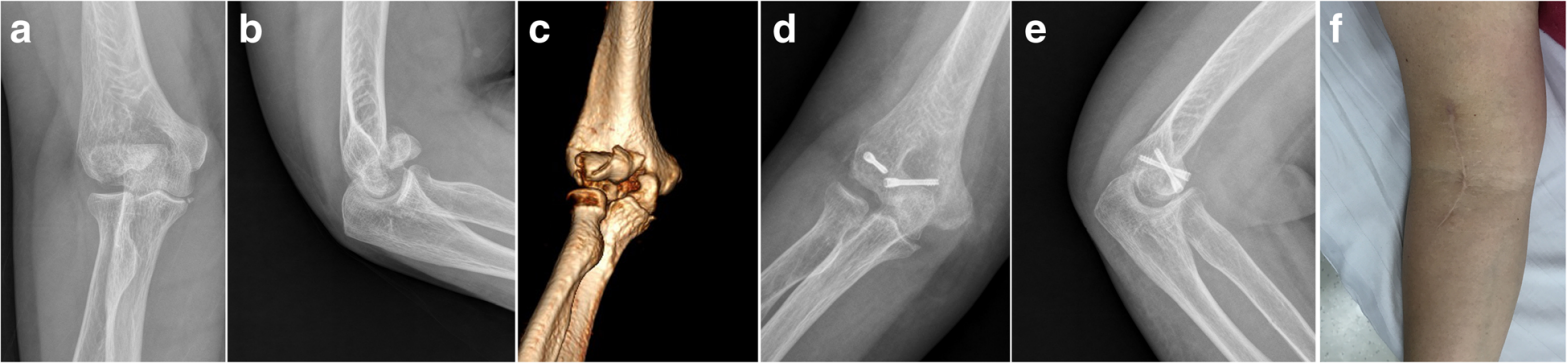
A 64–year-old female with type 3A capitellar fracture surgically treated with open reduction and Herbert screw fixation through anterolateral approach. **a** and **b**: Anteroposterior and lateral x-ray views of the fracture preoperatively. **c**: 3D CT reconstruction of the fracture preoperatively. The fragments were displaced anteriorly and superiorly. **d** and **e**: Anteroposterior and lateral x-ray views 12 months postoperatively showed union of the capitellar fracture which was fixed with 2 Herbert screws in an anterior to posterior fashion. **f**: Incision appearance of anterolateral approach

At the final follow-up, three patients reported mild pain and one described moderate ache during activity without restriction in activities of daily living. No patient had any subjective complaints of instability of the elbow. The average loss of ROM of the affected elbows was 10° of flexion-extension and 7° of supination-pronation compared with the unaffected elbows. But the average ROM of the affected and unaffected elbows did not differ significantly with respect to flexion-extension (134° ± 10°and 144° ± 4° respectively; *p* = 0.066), and supination-pronation (172° ± 11° and 179° ± 2° respectively; *p* = 0.083). The average MEPI Score was 93 ± 8 (range, 75 to 100) with 11 excellent and 4 good. All patients were satisfied with the operative outcome and returned to their previous activity levels. No evidence of avascular necrosis, posttraumatic osteoarthritis, or heterotrophic ossification was found (Table 1). An illustrative case was shown in Fig. 2.

**Table 1:** Demographics and clinical outcomes of reported patients

Patient	Gender	Age (year)	Mechnism	Dubberley classification	Follow-up (month)	ROM in flexion / extension (degree)	ROM in supination / pronation (degree)	MEPI Score
1	F	26	Fall	1A	24	145	180	100
2	M	48	Fall	1A	30	140	180	95
3	F	64	RTA	3A	36	130	160	85
4	F	35	Fall	1A	34	135	180	100
5	M	51	RTA	3A	28	125	155	85
6	M	28	Fall	1A	36	145	180	100
7	F	52	RTA	3A	31	120	155	85
8	M	19	RTA	1A	33	150	180	100
9	F	56	Fall	1A	29	130	175	95
10	M	60	Fall	1A	25	125	160	90
11	F	43	Fall	1A	24	130	175	95
12	F	39	Fall	1A	26	130	180	95
13	F	57	Fall	3A	27	120	155	75
14	F	33	Fall	1A	24	145	180	100
15	M	23	RTA	1A	24	145	180	100

*ROM* range of motion, *MEPI* Mayo Elbow Performance Index, *F* female, *Fall* ground level fall, *M* male, *RTA* road traffic accident

## Discussion

Surgical approaches are usually based on fracture type and complexity, comfort of the orthopedic surgeon, and protection of the blood supply. For coronal shear fracture of capitellum without posterior condyle involvement, the surgical approaches include the lateral approach and anterolateral approach to the elbow joint. The most commonly used approach in the literature is the lateral approach, which allows exposure to the elbow joint by elevation of the common extensor origin from the lateral epicondyle [2–8]. Nevertheless, its disadvantage is inadequate exposure of the capitulum and trochlea, which can limit visualization of accurate reduction of the fracture fragment, and make it relatively difficult to insert the screws perpendicular to the fracture site, especially for those capitellar fractures that extend to the trochlea. Dubberley et al. suggested that a flexor-pronator split or sectioning of the lateral collateral ligament should be performed if the medial aspect of the trochlea can not be seen adequately or reduction can not be confirmed from the lateral approach [2]. The anterolateral approach can expose the capitellum and trochlea widely enough to facilitate the reduction and fixation of the intra-articular fragments by directly approaching the anterior aspect of the elbow [9–11]. In addition, the anterolateral approach avoids the release of the common extensor origin, which may lead to postoperative extensor lag. So the use of anterolateral approach can circumvent the disadvantages of lateral approach. Based on our experience, the anterolateral approach was characterized by sufficient visualization of the joint including the medial articular surface, ease of achieving anatomic reduction and perpendicular fixation with screws in anterior to posterior direction.

The disadvantage for anterolateral approach is the plane of dissection much closer to the important neurovascular structures in the elbow, which carries a risk of iatrogenic injury to the radial nerve. In two research reports about anterolateral approach, no nerve injury occurred. Whereas Vaishya R et al. [11] reported one patient in their case series sustained postoperative posterior interosseous nerve palsy that recovered completely. In this case series, one incomplete posterior interosseous nerve injury occur, which recovered soon without residual compromise. In our opinion, the radial nerve dissected in this approach can be easily retracted and protected. The incidence of radial nerve injury is very small with direct visualization and careful retraction.

Fractures of elbow are usually associated with ligamentous injuries which can lead to elbow instability. Coronal shear fractures of the capitellum combined with elbow dislocation have been reported by Giannicola et al. as a potential pattern of complex elbow instability where eventual associated injuries of the lateral collateral ligament (LCL) and medial collateral ligament (MCL) should be assessed [13]. In the study of Mighell et al., Dubberley type 1A capitellar fractures were not associated with LCL injury, and two LCL injuries were found in five type 2A capitellar fractures [3]. Are A et al. reported no evidence of any LCL or MCL injury for isolated type 1A capitellar fractures [5]. In this series, no concomitant LCL or MCL injuries were found. In our opinion, ligamentous injuries occur in capitellar fractures combined with elbow dislocations. Examination under anesthesia should be done as a routine to rule out potential ligamentous instability. For coronal capitellar fractures with LCL injury diagnosed intraoperatively, lateral approach is preferred because the LCL injury and fracture can be treated through one lateral incision.

The method of fracture fixation is also an issue of interest for fracture management. Kirschner wires, metallic screws, bioabsorbable implants and fibrin glue have been reported for reconstruction of capitellar fractures [12]. Metallic screw fixation was the most commonly used technique. Biomechanical studies have demonstrated that metallic screws can provide favourable stability for constructed capitellum fractures [14, 15]. Although several different types of screw (cannulated, cortical lag, cancellous, headless, and Herbert screws) were used, no direct comparison between the different screws could be made due to the heterogeneous reporting of clinical outcomes. Nowadays, Herbert screws fixation has become popular for capitellar fractures and good clinical results have been published because the advantages offered by these screws include excellent compression at the fracture fragments, stable fixation, and nonprominence of the implant intraarticularly [7, 16].

In the present study, good-to-excellent outcomes were achieved according to MEPI Score for all the shear fractures of capitellum without involvement of posterior aspect. These results were similar to those previously published [4, 6, 17]. Although four patients reported mild or moderate pain during activity, all patients were satisfied with the operative outcomes and returned to their previous levels of activity. Furthermore, no avascular necrosis or posttraumatic osteoarthritis occurred in this case series, which is in accordance with several studies [5, 7, 9–11]. In our opinion, protection of capitellar soft tissue attachment, anatomical reduction and stable fixation with minimal damage to articular cartilage can minimize the incidence of both complications. In general, satisfactory outcomes for coronal shear fractures of capitellum can be expected by accurate reduction, adequate fixation and early functional exercises. All of these can be easily achieved by the use of Herbert screw fixation through anterolateral approach to the elbow joint.

The limitation of this study was the small number of patients and short-term follow-up period. The larger numbers of patients and longer follow-up period is necessary to determine the true incidence of osteonecrosis and posttraumatic arthritis.

## Conclusion

Our findings suggest that open reduction and Herbert screw internal fixation through the anterolateral approach is a reliable and effective treatment for coronal shear fractures of capitellum, and able to achieve stable fixation and restoration of a functional range of motion.

## Abbreviations

LCL: Lateral collateral ligament
MCL: Medial collateral ligament
MEPI: Mayo elbow performance index
ROM: Active range of motion
